# Navigating Services in the UK: The Lived Experiences of Families Affected by 22q11.2 Deletion Syndrome

**DOI:** 10.3390/bs16010019

**Published:** 2025-12-22

**Authors:** Maria Gudbrandsen, Sophie Edmonds, Michelle Jayman

**Affiliations:** 1 Centre for Research in Psychological Wellbeing (CREW), School of Psychology University of Roehampton, London SW15 4JD, UK; michelle.jayman@roehampton.ac.uk; 2 NHS Surrey and Borders Partnership, Leatherhead Surrey KT22 7AD, UK; sophie.edmonds@sabp.nhs.uk

**Keywords:** 22q11.2 deletion syndrome, participatory action research, mental health and wellbeing, transition to adulthood, family advocacy, service gap, lived experience, holistic care models

## Abstract

Families affected by 22q11.2 deletion syndrome (22q) face complex health and mental health challenges, yet their lived experiences, particularly within the UK, remain underexplored. This study aimed to understand how families navigate care systems, mental health provision, and the transition to adulthood. Using a participatory action research (PAR) framework, five young adults with 22q and six parents were interviewed, with a steering group co-developing the research questions to ensure relevance and accessibility. Thematic analysis revealed five key themes: lack of professional awareness and diagnostic delays; fragmented and generic care pathways; emotional burden of parental advocacy; systemic gaps during transition to adulthood; and the enabling role of supportive relationships and environments. These experiences highlight a need for holistic, collaborative models of care, improved professional training, and inclusive support systems tailored to the unique needs of individuals with 22q. By centring family voices, this study offers critical insights into systemic barriers and facilitators in the UK, with implications for policy, practice, and future research.

## 1. Introduction

22q11.2 deletion syndrome (22q) is a complex genetic condition affecting approximately 1 in 2148 live births (Blagojevic et al., 2021). It presents with a wide range of physical, cognitive, and psychiatric manifestations, including congenital heart defects, immune dysfunction, learning disabilities, and increased risk of mental health conditions such as anxiety, depression, and psychosis (McDonald-McGinn et al., 2015; Schneider et al., 2014). Individuals with 22q have been reported to have lowered resilience and quality of life compared to both the general population and those with intellectual disabilities; with complex health challenges significantly affecting their overall wellbeing (Van de Woestyne et al., 2022). While the clinical variability of 22q is well documented, families navigating 22q often face systemic barriers that extend beyond medical care, shaping emotional wellbeing and everyday life. Existing research highlights fragmented care pathways and limited professional awareness, yet the lived experiences of families, particularly within the UK context, remain underexplored. International studies have identified gaps in mental health provision and transition planning, but few have examined how these challenges intersect with the impact of advocacy by families, nor have they examined the resilience during critical life stages such as the move to adulthood (Ayoub et al., 2024; Gadancheva et al., 2023). Given the high prevalence of 22q, addressing these gaps is critical to inform UK healthcare policy and practice. Insights from lived experience can guide the development of more responsive, integrated services and targeted interventions.

### 1.1. Physical and Mental Health Challenges

For context into the barriers experienced by families, it is important to first note the quite complex health and mental health difficulties experienced by this population. For example, individuals with 22q experience a wide range of health complications that typically require lifelong, multidisciplinary care, with congenital heart defects among the most frequent and clinically significant features (McDonald-McGinn et al., 2015; Óskarsdóttir et al., 2023). Palatal anomalies, often contributing to feeding and speech difficulties (Campbell et al., 2024), immunodeficiency, endocrine disorders, and gastrointestinal disturbances are amongst other common symptoms affecting these individuals (McDonald-McGinn et al., 2015). Throughout the literature, it is clear that these multisystem challenges highlight the importance of early diagnosis, regular monitoring, and coordinated care to support health and development across the lifespan (Óskarsdóttir et al., 2023).

Individuals with 22q are at an elevated risk for a range of mental health conditions across the lifespan. Anxiety and mood disorders are frequently observed, with anxiety often presenting early and mood disturbances appearing later in development (Fung et al., 2010). Neurodevelopmental conditions such as autism and attention-deficit hyperactivity disorder (ADHD) are common and often co-occur with other psychiatric diagnoses, complicating clinical presentation and functional outcomes (Schneider et al., 2014). Subthreshold psychotic symptoms can emerge during adolescence, and the risk of developing schizophrenia or related disorders in adulthood is notably high, positioning 22q as one of the most significant genetic contributors to psychosis (Provenzani et al., 2022; Schneider et al., 2014; Tang et al., 2014). These overlapping challenges can significantly affect cognitive, emotional, and social functioning in 22q, and current clinical guidelines emphasise the importance of early identification, continuous monitoring, and integrated care approaches that address both psychiatric and psychosocial needs (Campbell et al., 2024; Óskarsdóttir et al., 2023; Van de Woestyne et al., 2022).

### 1.2. Families Navigating Barriers and Care Pathways

Despite the complex medical and psychiatric difficulties experienced by 22q, studies conducted internationally have identified that health professionals working in local (mainstream) mental health services have limited knowledge and understanding of 22q and associated conditions (Carrion et al., 2022). Families affected by 22q often face significant emotional, practical, and systemic challenges that permeate daily life. Delayed or missed diagnoses are common, often resulting from the condition’s variable presentation and limited awareness among healthcare professionals; both affect early access to appropriate interventions, as well as contribute to parental frustration and uncertainty (Ayoub et al., 2024; O’Donoghue et al., 2022). Even after diagnosis, families frequently encounter fragmented healthcare systems, characterised by poor communication between services and a lack of coordinated care pathways (Vo et al., 2018). Parents report feeling dismissed or misunderstood by professionals, despite their extensive knowledge of their child’s needs (Goodwin et al., 2020), and the lack of recognition and collaboration exacerbates the emotional burden on families, fostering a pervasive sense of isolation and exhaustion. Often, the lack of coordinated care and tailored support services contributes to a pervasive sense of isolation and emotional exhaustion (Goodwin et al., 2020; Vo et al., 2018).

In addition, despite the well-documented association between 22q and elevated psychiatric vulnerability in terms of mental health, support here is often considered lacking or inadequate. Access to mental health services for individuals is limited by several barriers that impact both diagnoses and ongoing care, affecting overall wellbeing. For example, healthcare providers lack awareness of 22q and its psychiatric risks, often leading to under-recognition and delayed diagnosis of both the syndrome and associated mental health conditions (Abu-Ghname et al., 2020). Families also frequently report that mental health services are not well equipped to meet the complex needs of individuals with 22q, and existing interventions are described as superficial, generic, and lacking continuity, with professionals demonstrating limited awareness of the syndrome and its psychiatric implications (Goodwin et al., 2017; Goodwin et al., 2020). Families experience long delays in accessing specialist mental health support, compounded by fragmented care pathways and poor communication between services (O’Donoghue et al., 2022). A recent review found that individuals with 22q identify mental health challenges as among the most significant barriers to quality of life yet report services as inaccessible and poorly adapted to their cognitive and emotional needs (Ayoub et al., 2024). Furthermore, very few specialist 22q services exist, and these are typically not located in the same area that the person lives and rarely provide ongoing mental health support (Campbell et al., 2024). The emotional toll of persistent advocacy, combined with systemic resistance, frequently leads to strained family relationships, internalised guilt, and burnout (Goodwin et al., 2017). Combined, these suggest a systemic failure to provide timely, tailored, and holistic mental healthcare for this population, leaving families to navigate a complicated system.

While mental health challenges persist across the lifespan, they become particularly acute during the transition to adulthood, a period marked by systemic discontinuity, heightened vulnerability, and shifting expectations that profoundly shape the lived experience of young people with 22q and their families (Gadancheva et al., 2023).

### 1.3. Transition to Adulthood and Lived Experience

Transitions to adulthood represent a high-risk period for young people with 22q and their families, often described as one of the most challenging periods in the life course (Kerin et al., 2020; Phillips et al., 2017). This stage is marked by sudden changes in healthcare provision, including the shift from paediatric to adult services, which is frequently poorly coordinated and lacking in continuity, as well as poor communication between providers (Ayoub et al., 2024; Gadancheva et al., 2023). Lack of standardised transition protocols and resources for adolescents and adults with 22q further hinders access to ongoing mental health support (Fung et al., 2015; MacIsaac et al., 2025).

Families report that the loss of familiar routines and trusted professionals, combined with the pressure to conform to adult expectations, can trigger significant emotional distress for both young people and their caregivers (Goodwin et al., 2020). Parents express heightened anxiety about their child’s future, especially regarding independence, financial security, and access to appropriate mental health and social support (Vo et al., 2018). These concerns are compounded by systemic gaps in transitional planning and the absence of tailored pathways that address the complex interplay of physical, cognitive, and psychiatric needs associated with 22q (Ayoub et al., 2024; Kerin et al., 2020). Together, these findings underscore that transition to adulthood for individuals with 22q is not only a developmental milestone but also a critical juncture where systemic discontinuity and inadequate planning intensify emotional vulnerability.

### 1.4. Addressing Systemic Gaps

Although previous research has provided valuable insights into the psychosocial impact of 22q on families (Goodwin et al., 2020; O’Donoghue et al., 2022; Vo et al., 2018), significant gaps remain. Evidence on transitional planning, continuity of care, and mental health provision largely reflects international contexts, with little attention to the UK healthcare system and its unique structural challenges. Furthermore, most studies often rely on retrospective accounts and parental perspectives, overlooking young people’s voices (Ayoub et al., 2024), with few adapting participatory approaches, actively involving families in shaping research priorities.

This study addresses these gaps by adopting a participatory action research (PAR) framework, centring parent and young adult perspectives to explore the lived experiences of navigating health and mental health support, as well as transition to adulthood. The study aims to offer UK-specific insight into the systemic barriers, facilitators, and experiences of families affected by 22q. By contrasting these findings with existing 22q research internationally, we seek to highlight unique challenges to UK healthcare systems. Prioritising the voices of those who matter the most, this research seeks to inform responsive, holistic approaches to care for 22q.

## 2. Materials and Methods

### 2.1. Design

This study adopted a PAR framework to ensure individuals with lived experience of 22q were actively involved in shaping the research process and outcomes, grounded in principles of equity, collaboration, and empowerment (Koch & Kralik, 2006). It recognises and prioritises the unique experiences of this population in issues that affect them directly, aiming to generate knowledge that supports social justice and meaningful change related to mental health and access to healthcare (Macdonald, 2012), as well as support a flexible, iterative approach to data collection and interpretation. A steering group, comprising a young person with 22q and their parent dyad, as well as a mental health practitioner with 22q experience, was actively involved in shaping the research design by co-developing the interview schedule. This was to ensure that questions were relevant to the 22q population and reflective of concerns the families may have.

### 2.2. Participants

A total of five young adults with 22q and their parents (all mothers) took part, forming five dyads, as well as one additional parent participant of a 31-year-old daughter (Cara’s parent). Participants were recruited through purposive sampling, with calls for expressions of interest disseminated via relevant social media and third-sector organisations, and screened to ensure they met the inclusion criteria (a confirmed 22q diagnosis) and exclusion criteria (severe learning difficulties and/or current psychotic symptoms). All participants were white British. The sample size is consistent with PAR designs, prioritising depth and co-production over breadth if data is sufficient in achieve thematic saturation (Braun & Clarke, 2006; Swain, 2018). Pseudonyms were used throughout to protect participant confidentiality (see Table 1 for young adult characteristics).

### 2.3. Procedure

Ethical approval for this study was provided by the University of Roehampton ethics board (PSYC 22/424). This study was conducted in two stages in accordance with PAR principles and the affirmative framework. Stage 1 involved an online steering group to co-develop the research protocol, ensuring stakeholders were involved in the research process to accommodate the different developmental needs and capacities of the 22q population. This process shaped validity by ensuring questions reflected lived priorities and were accessible. The steering group comprised one young person–parent dyad, one mental health practitioner with extensive 22q knowledge, and Authors 1 and 3; the interview was audio recorded, lasting a total of 90 min. This informed the content, structure, langue, and accessibility of the interview guide, including visual prompts and vignettes. The authors acknowledged the need for flexibility, adapting methods and tools to suit participants and recognising that engagement with young adults was inherently fluid, negotiable, and unpredictable (Madge et al., 1997).

The knowledge production phase, Stage 2, was facilitated by Authors 1 and 3 and involved semi-structured interviews conducted online (developed from Stage 1), used to allow for the uncovering or expansion of information that held significance for the participant (Gill et al., 2008). The authors remained reflexive throughout both phases and conscious of disparities in power and status between the adults (researchers and parents) and young people, and they employed strategies to address this. For example, regular member checking was performed to prioritise CYP voices throughout. This collaborative design strengthened ecological validity and aligned with PAR’s ethos of equity and empowerment. The interviews lasted between 45 and 90 min, with breaks as requested, and were audio recorded. The semi-structured guide included questions largely across three key topic areas: awareness and context, support and barriers, and transitions and communication. See Table 2 for example questions.

All the young adults with 22q opted to have their parent present during their interviews; however, their voices were prioritised throughout. All parent interviews were conducted separately on the same day. Each participant received GBP 30 as thanks for their time and contribution. Interview recordings were transcribed verbatim by Author 2.

### 2.4. Data Analysis

A hybrid thematic analysis (TA) was employed to explore the participants’ experiences, combining deductive and inductive strategies. This method was selected due its flexibility and suitability for participatory research, as it allows for the integration of lived experience with theoretical insight (Proudfoot, 2023; Swain, 2018). The overarching research question, examining barriers and facilitators to health and mental health provision, was informed by the existing literature, providing a conceptual lens that guided the analysis. See Table 3 for examples.

With the interview questions being informed by a stakeholder steering group, a participatory and exploratory element was added, allowing for the inclusion of perspectives not directly driven from theory. During the analysis stage, themes were allowed to emerge organically from the data while remaining sensitised to the guiding concept of barrier and facilitators. This approach enabled a flexible yet theoretically informed interpretation of the data, consistent with a hybrid analytical framework and complimentary to the PAR ethos of the study, ensuring that the voices of young adults with 22q and their families were central to the interpretation and representation of the findings (Swain, 2018). While themes were developed through the hybrid inductive–deductive approach, participant voices guided interpretation, and reflexivity and co-production were maintained throughout to ensure findings were grounded in lived experience rather than researcher assumptions.

The analysis followed Braun and Clarke’s six-phrase framework (Braun & Clarke, 2006), adapted through the hybrid approach proposed by Swain (2018) via the below steps (Table 4).

A calibration exercise was conducted on one transcript to align coding strategies. All transcripts were coded independently by Authors 1 and 2, with discrepancies resolved through discussion. Author 3 reviewed the themes for coherence. Reflexivity was maintained through reflective memos and discussions, ensuring interpretations remained grounded in participant narratives. These steps enhanced reliability and transparency in the analytic process. Credibility and confirmability were supported through thick description and the inclusion of exemplar quotes for each theme and subtheme. Quotes were selected to illustrate the diversity of perspectives and maintain authenticity of participant voice.

## 3. Results

**Figure 1:**
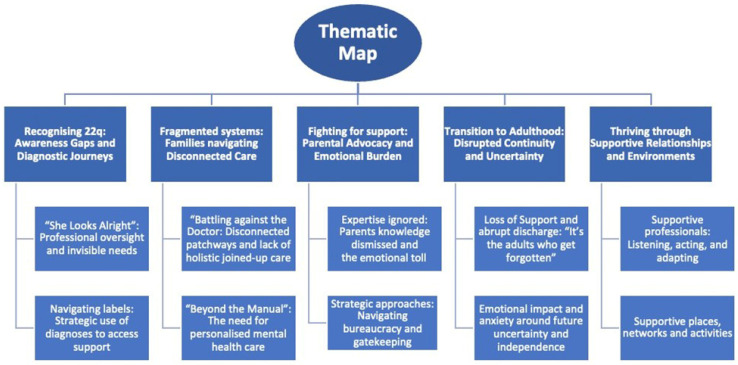
Thematic map illustrating five superordinate themes and associated subthemes derived from the thematic analysis.

The analysis generated five superordinate themes that captured families’ experiences of navigating health and mental health support for young people with 22q in the UK, including experiences of transition. These themes reflect both systemic barriers and enabling factors. Most themes illustrate how fragmented care pathways, rigid mental health provision, and abrupt transitions interconnect with advocacy burden and emotional fatigue. At the same time, families identified relational and environmental supports that fostered resilience and wellbeing. See Figure 1 for a thematic map illustrating emergent themes and subthemes capturing families’ experiences.

### 3.1. Theme 1. Recognising 22q: Awareness Gaps and Diagnostic Journeys

This superordinate theme captures challenges faced by families around the lack of recognition of the 22q condition amongst professionals. It explores how lack of awareness, reliance on visible disability markers, and strategic use of alternative diagnoses influence pathways to support.

#### 3.1.1. “She Looks Alright”: Professional Oversight and Invisible Needs

One of the most prominent barriers to support for individuals with 22q was the widespread lack of awareness of 22q amongst health professionals. Parents frequently described encounters with practitioners who had never heard of the 22q condition or simply dismissed its relevance: *“Most professionals don’t know what it is”* (parent of Sarah) and “*with 22Q you say that to most people… they’re like, what’s that? (parent of Maggie)*. Some professionals had heard of it but were not sure what it entailed. Often this was compounded by the fact that individuals with 22q do not always present with physical characteristics, leading to assumptions that they are ‘fine’ or not in need of support. As Sarah’s parent explained: “*Unless you’re walking in there with a disability that everyone can see… she looks alright*.” Cara’s parents reflected on a SENCO during her time at school who said: *“I’ve got to go now because I’ve got to go and deal with the child who’s got no legs. He’s got real needs.”*

This lack of recognition often delayed diagnosis and intervention, as well as led to feelings of being dismissed or ignored, even when families raised concerns repeatedly. Several families described long and frustrating journeys to diagnosis, often only achieved after persistent advocacy or by chance encounters with more knowledgeable clinicians. Thomas’s mother explained that she had a *“stand-up row”* with a hospital doctor before finally being referred to a consultant, who then recognised the signs of 22q and the need for genetic testing. She described how much of a relief it was, to finally *“get a doctor that was actually listening to us.”* Anna’s mum echoed this as she had *“been asking for help ever since she was a baby”.* Other parents ended up paying for private care to receive help, and Peter’s mum explained that this sped things up significantly and that the ENT [ear, nose and throat] doctor looked in his mouth and *“virtually straight away said, I don’t think you’re prepared for what I’m seeing [*i.e., *a diagnosis of 22q].”*

#### 3.1.2. Navigating Labels: Strategic Use of Diagnoses to Access Support

In the absence of 22q recognition, families often found themselves relying on more familiar diagnoses such as autism or anxiety disorders to access needed support. Sarah’s mum shared that she would often just tell people her daughter had autism rather than both 22q and autism, as with 22q, *“people go, that’s nice. What’s that?”*. Maggie’s mum did something similar, as people had a better understanding of more familiar labels.

The process of securing additional diagnoses was frequently described as a bureaucratic necessity rather than a reflection of the child’s full experience. Parents like Sarah’s mum expressed frustration that 22q alone was not sufficient to trigger support, despite its wide-ranging impact, and she needed to instead rely on her autism diagnosis.

*“And it is a label that gives you opportunities. Really, it’s not a label that you want, it’s a label that you need. And that’s the sad thing, isn’t it, that you want your child to be diagnosed with autism because it means that you can access help. With 22Q11 you can’t. It doesn’t give you anything.”*

Peter noted, *“With 22Q it’s like an umbrella. I think you have bits of everything.”*

Similarly, families reported being frequently met by barriers when seeking needed medical help. As an example, upon trying to secure help for her chronic fatigue, Sarah encountered a very dismissive physiotherapist who said to her mum, *“how many diagnoses do you want?”*

These experiences reveal that while recognisable diagnostic categories may allow for access to further support, this is not often the case for 22q, forcing families to navigate a complex and emotionally fraught landscape of labels to secure help. Often this more strategic use of labelling was necessary but sometimes came at a cost. While some parents viewed a diagnosis as a necessary gateway to support, others expressed concern about the psychological impact of labelling on their children. Anna’s parent reflected on the harm caused by a later diagnosis of autism, describing how her daughter internalised it as *“another thing that’s wrong with me.”* Maggie said having 22q is harder *“when people don’t understand it… I’m just [M] and that is the way it is”.*

This superordinate theme highlights how limited professional awareness and diagnostic delays shape families’ strategies for accessing care, underscoring the systemic barriers to timely recognition and intervention.

### 3.2. Theme 2. Fragmented Systems: Families Navigating Disconnected Care

This superordinate theme captures the often-disjointed nature of health and mental health services, where families had to act as care coordinators, often intensifying stress and creating barriers to holistic, joined-up care.

#### 3.2.1. Battling Against the Doctor: Disconnected Pathways and Lack of Holistic, Joined-Up Care

When families did receive their diagnosis, often after fighting for it, many still reported feeling abandoned or misunderstood by services: *“We just felt we were battling against the doctor”* (parent of Thomas)*,* particularly within mental health and psychiatric care: *“Even now… the psychology unit that we’re waiting for, they’ve not heard of 22q”* (Cara’s mum). The lack of coordination, empathy, and specialist knowledge among professionals contributed to a sense of frustration, helplessness, and emotional exhaustion. Families described a lack of well-rounded care where services often operated in silos, with little communication between professionals across sectors, leaving parents to coordinate care themselves: *“I think a lot of specialities are just concentrated on their speciality”* (parent of Maggie).

While parents understood that services were likely overwhelmed and professionals see many patients, they felt their interactions with patients and families could be much better and, at times, often felt overwhelmed by the sheer volume of information. Maggie’s mum reflected on previous appointments where, referring to the doctor, “*they didn’t actually stop to take breath when they were … so before you had time to think about the answer to one thing and moved on to the next. And I struggled… It sounds awful, doesn’t it? But it is very much like a conveyor belt”.*

Families encountered a disjointed and under-resourced system of care. Due to the often complex medical and mental health difficulties, they often had to attend a number of different hospitals across the country and liaise with several services and departments. Families expressed a real need for a single point of contact or care coordinator to help navigate the complex web of services spread across multiple hospitals and specialities. Maggie’s mum explained, *“Because it is so fragmented over so many different hospital… if there was at least somebody that you could go to, a care coordinator, so that you can then say, can you help support with this?”*

Families also felt dismissed and unheard by professionals, and some described a lack of empathy or respect, compounding their stress and undermining their confidence in the systems. Some parents have addressed this directly, like Maggie’s mum: “*I have actually said something to a couple of consultants, you know, actually she’s a person. I’m a person. So at least be a bit more respectful… she was petrified*”. Cara’s mum added that, *“Sometimes they forget to treat every child as an individual, don’t they?”,* and *“They don’t really get it… they don’t seem to appreciate the 22q.”*

#### 3.2.2. “Beyond the Manual”: The Need for Personalised Mental Healthcare

Families described inadequate mental health services, especially Child and Mental Health Services (CAMHS) and psychiatric care more broadly as dismissive and underprepared, as indicated by Cara’s mum: *“The psychiatric care… I just think is appalling.”* Families often experienced advice as superficial or inappropriate, and support lacked continuity or follow-through. One parent noted that when her daughter suffered severely with suicidal thoughts: *“It was very dismissive to the point where it was like, hide the knives. Just hide them. It’ll be fine. Just hide the knives. And that’s what that’s the advice I was given.”*

Peter noted, *“I guess the mental health unit, you know, in our place in our hospital and they’re not in the best”,* while his mum described being *“disillusioned with the psychiatric ward”*. Peter explained that his former [private] psychologist had *“printed off stuff about 22q for me and I showed it to one of the mental health nurses and she didn’t want to know*.”

Services were also often described as rigid and generic, and often not suited to the complex and variable needs of young people with 22q. Interventions were often “*tick-box*” exercises, with little room for individualisation or adaptation. There were few attempts to spend time engaging the young person in treatment if they did not respond as expected, with some moving through several unsuccessful therapies, as explained by Anna’s parent: *“[She] needs to be able to experience it from a safe space before she’s ready to engage… but then we were being told that [it] was a failure.”* Anna and her mum felt that CAMHS had a “*poor understanding of her needs.”* In one session, Anna noted, *“They just got a bit impatient with me not trying as they wanted us to mix straight away with other people”* and she was unable to due to her severe anxiety. Her mum elaborated that they *“basically told her that she wasn’t trying hard enough, but the fact that she was even in that room, with other people for [Anna], was a big try*”. In the end, Anna just stopped going.

There was a particular lack of awareness of some of the severe mental health outcomes for 22q like psychosis. Sarah’s mum noted, *“It’s them not listening to the parent saying this is what’s going to happen if we don’t do this… and it’s sort of like fallen on deaf ears”.* With episodes of psychosis being common amongst adolescence and early adulthood in 22q, it can have a big impact on them as they have to deal with *“quite a scary kind of thing”.* Parents felt that something should be in place to support them and explain it to them: *“Maybe somebody that they can speak to if they’ve got concerns… like you have a play specialist for surgery, but for the older ones, it’s probably not so”* (parent of Maggie). She noted, *“In a way you can understand rather than, you know, sort of stepping into this black hole of somethings going to happen, but we don’t know what or how or when. And so not really having that understanding.”*

However, when young people came across professionals who took the time to learn about 22q, it made a significant difference. Peter paid to see a private psychiatrist, who took the time to learn about 22q and treated it accordingly. He noted that this “*finally changed things”,* and his mum added that it really *“changed his life.”* Cara also found a psychiatrist who made a big difference, and her parent reflected that “*she listened to us as parents as well as [Cara].”*

This superordinate theme demonstrates how fragmented systems and a lack of coordination force families to act as care coordinators, revealing structural gaps that hinder holistic and continuous care.

### 3.3. Theme 3. Fighting the Support: Parental Advocacy and Emotional Burden

Parents of children with 22q often find themselves in the dual role of advocate and caregiver, navigating a system that has a lack of awareness of the 22q condition, and frequently ignores their expertise, failing to provide adequate support. This superordinate theme reveals the emotional weight of parental advocacy, the frustration of being dismissed, and the transformative impact when professionals listen and adapt.

#### 3.3.1. Expertise Ignored: Parents’ Knowledge Dismissed and the Emotional Toll

Despite their deep understanding of their child’s needs, parents often encounter professionals who dismiss their insights or rely on rigid, generic approaches. This lack of collaboration leads to frustration and delays in care. Peter’s mum reflected, *“They just see everyone on the same like manual… like they read from a book”.* Sara’s mum found that “*It was like banging your head against a brick wall… I was having to take people with me so that they would listen*”, and Thomas’ mum added, *“They should be led by the parent and just listen and think, OK, maybe there is something in what they’re saying.”*

Even when additional information was available to help professionals, this was still ignored, as illustrated by Anna’s mum: *“Clinical psychology did a lot of work… they put together a passport that explained everything [Anna] needed… and they [other services] still didn’t really do anything.”*

The advocacy journey was emotionally draining for most of the families. Parents describe frequent tears, feelings of helplessness, and the psychological toll of constantly fighting for their child’s needs. Sarah’s mum reflected, *“I spent a lot of my time in tears in these appointments. And my husband just throws me the tissue”,* and *“It’s not nice sitting there fighting these battles on your own”.* Sarah’s mum further explained that after years of these challenges, “*You sort of get a bit hardened to it… You shouldn’t have to… but as a parent you sort of have to*.”

The emotional toll extended beyond the individual parent to the entire family unit. Parents describe disrupted relationships, guilt over decisions made under pressure, and the impact of caregiving on siblings and partners. Parents described how the constant vigilance, advocacy, and emotional labour required to support their child led to strained relationships, disrupted routines, and feelings of guilt, both for what they could not do and for the impact on other family members. Some parents internalised blame for their child’s struggles, especially when professionals failed to recognise the syndrome or offered inadequate support. This guilt was compounded by the pressure to make difficult decisions, such as pursuing diagnoses, pushing for services, or considering supported living arrangements, without clear guidance or reassurance. Cara’s mum reflected, *“Sometimes I look back and think I failed her… But at the time you do what you’re told.”*

Due to the lack of awareness, despite a clear link between 22q and mental health difficulties, including episodes of psychosis, families encountered blame. Cara’s mum found that *“Unfortunately, when we first started trying to access mental health… they kept blaming us as parents. The parenting… And I kept saying to them, but I’ve got two older daughters who are perfectly fine….”* Sarah also reflected on this and explained, “*And [Sarah] is our fifth child… So, it’s not as though we wasn’t experienced, it was just that it was a whole new, different ballgame.”* For Cara’s family, the strain manifested in family dynamics, particularly with siblings, as it inadvertently caused harm, placing blame on the family unit: *“They dragged the family into family therapy… it absolutely destroyed my relationship with my two [other] children.”*

#### 3.3.2. Strategic Approaches: Navigating Bureaucracy and Gatekeeping

While the parents of the children with 22q often demonstrated extraordinary commitment, their advocacy was frequently not a choice but a necessity and a prolonged and emotionally exhausting battle. The parents described how they were forced to navigate a fragmented and unresponsive system, where their concerns were frequently dismissed, their expertise overlooked, and their children’s needs misunderstood or minimised.

Rather than being met with collaborative care, the parents often encountered gatekeeping, bureaucracy, and a lack of professional interest. This meant that even when a diagnosis was in place, services were not automatically triggered. Instead, families had to push, challenge, and escalate, sometimes repeatedly, just to access basic support. Cara’s mum noted that *“It took a long time to get the right treatment because we had to work with the old team… who weren’t open to us suggesting things.”*

Some families had to ‘fight’ not just for medical services but also for other needed services. For one family, this was to access residential support as their daughter got older. Maggie’s mum shared that, just to acquire the support needed, *“We had to take the local authority to court… it was immensely stressful.”* For Cara, her parents had *“to complain to the head of speech and language… it wasn’t easy coming.”* Others, like Peter’s mum, felt that it was *“like lip sync… they were saying it while you were there, but there was nothing following on.”*

Often, because of systemic shortcomings, families were forced to seek private care, travel long distances, or engage in legal processes for care that should have been the standard. These additional efforts came at significant emotional, financial, and relational costs, often exacerbating stress already experienced as caregivers and advocates. Sarah’s mum ended up paying privately to secure much needed care for her daughter on more than one occasion: *“In the end we paid privately to get her seen by a rheumatologist… She was literally in bed 24 h a day. This is ridiculous.”* And when she was still at school, she *“got an educational psychology report done privately because she had only been seen by an educational psychologist through the local authority twice… There was nothing in between.”* Peter ended up seeing a private psychiatrist after his experiences. Families frequently drove long distances to attend multiple specialist clinics, which was *“horrendous”,* despite service *“saying it should be provided locally.”*

This superordinate theme shows how persistent advocacy becomes an emotional and practical burden for parents, illustrating the cost of systemic resistance and the need for collaborative approaches.

### 3.4. Theme 4. Transition to Adulthood: Disrupted Continuity and Uncertainty

Transitions to adulthood were an emotionally and practically challenging phase for individuals with 22q. This superordinate theme captured the critical point where systemic discontinuity collided with heightened vulnerability.

#### 3.4.1. Loss of Support and Abrupt Discharge: “It’s the Adults Who Get Forgotten”

Families consistently found the transition from paediatric to adult services abrupt and poorly managed, often coinciding with critical mental health needs. The withdrawal of support was sudden and unprepared, leaving families to navigate complex challenges without guidance or continuity. As noted by Cara’s parent, *“And then when she got to 18, I went into one appointment, and they said ‘Oh she’s being discharged, and she’s got to go and find a job.’ And that was it. No continuing care.”*

While there has been an increase in available information about 22q, most has been aimed at understanding children, and often adults are forgotten. This is especially true for the clinics available to 22q adults. Peter explained, *[There is] more stuff when we’re adults as well, because that’s what annoys me. Like when you’re a kid, you get all their support… But then even as an adult, you could need stuff as well. And it’s obviously not much for that”.* Sarah’s parent agreed that there was a lack of adult services for 22q and added, “*it’s the adults who get forgotten… I think sometimes in adolescence and that’s what your troubles start. That’s where our troubles started, we were fine up until that point…”* The transition to adult services was further marked by a decline in specialist care and understanding. Family and adult services were generic, rigid, and ill-equipped to accommodate the complex needs of individuals with 22q. As expressed by Sarah’s parent, *“It’s adult centres, and that’s your generic thing. So, it’s generic services wherever you go…. Whereas when you go to Great Ormond Street [hospital] it’s a very nurturing environment and a very kind environment. you then just get stuffed in with the rest.”*

#### 3.4.2. Emotional Impact and Anxiety Around Future Uncertainty and Independence

The pressure to conform to adult expectations, employment, independence, and social integration was overwhelming and distressing. Families reported that these expectations often triggered emotional deterioration, particularly when young people were not developmentally ready. The developmental gap between those with 22q and their peers becomes increasingly larger as they grow older.

Cara’s parent reflected that she was just not ready to think about the future at the stage it was expected of her (end of year 9 and 10) and “*that just freaked her out… And that’s when it all started [mental health issues].”* Sarah had a similar experience: *“In the last year of school, it did get quite bad, quite challenging… So, you’re looking at colleges with her… she knows she’s not going to go with any of the friendships that she’s made… it got worse.”*

Parents also expressed deep concern about their child’s ability to manage adult responsibilities, particularly in the absence of parental support. These concerns were often compounded by their concerns around independent living. The majority of the young people still lived at home with their parents, and one was in supported living. Cara’s mum in particular reflected, *“She’ll paint this picture of a really happy and independent [Cara], when you know that she can’t even have a shower on her own because she won’t dry properly, you know…”* Maggie is in supported accommodation, but that was only an option for her after her parents fought very hard for that to happen. Parents also worried about the overall independence of the young person, whether financial or emotional. Peter’s parent noted, *“I worry that when I’m not around and when his dad’s not around… how he would manage? Dealing with bills and finances and needing reminders for certain things in his life.”* Peter interjected, “*Because I’m terrible at that now*”, and mum agreed, “*he does need a lot of prompting on things*.”

While Thomas’ mum expressed that he was doing well for himself and managing parts of his finances himself, he still lived at home and his dad sat with him every month when he got paid in order to “*work his finances out with him”.*

Cara’s parents had recently started the process of securing supported living for Cara, despite knowing it would cause her severe anxiety to build up, as they recognised she would not be able to live with them forever: “*I didn’t think it was fair to expect either one of her sisters to take her on when we can’t no longer do it. Now obviously that has increased her anxiety…. So obviously she’s going to need a really high level of care, …. I just feel I’m not getting younger.”*

Despite some positive reflections, parents continued to express deep worry about their child’s future. These reflections captured the enduring emotional weight of caregiving and the persistent anxiety about what would lie ahead. *“I think it was when [Peter] was younger… I was worried. I was fearful for his future… I [still] do worry about him now”.* These reflections underscore the persistent anxiety parents feel, even when their children show signs of progress. The emotional labour of caregiving does not end with adulthood, and the lack of systemic support leaves families carrying the burden alone.

While both Thomas and Peter had jobs, this had often come at a cost. Previous jobs resulted in a deterioration of their mental health if they were not properly understood and supported. Peter’s parent reflected on his job as a care worker, stating that “*It was getting too much for him… they were always calling him because he always said yes, you know [on his] days off he’d go in. He was really burning too much… And I think it took its toll and the depression come out massively.”*

Thomas had a similar story after getting a job in a pub soon after college. His parent explained, “*I picked him up [after the first day] and he cried. Quite a bit when [we] got home because he just didn’t want to go back… And I knew it was the anxiety. It was just being in an environment that he wasn’t familiar with”.*

Both young men were currently unsure of the future. Peter reflected, “*I’ve got ambition… but I don’t know what I want to do*”, and Thomas noted, *“I just crack on with work and stuff. I don’t really have any [plans] to be honest. I just do one day at a time.”*

This superordinate theme captures how abrupt transitions and future uncertainty amplify vulnerability, emphasising the importance of structured planning and adult-focused services.

### 3.5. Theme 5. Thriving Through Supportive Relationships and Environments

This final superordinate theme highlights the positive experiences for families, including supportive professionals, networks, and environments, as well as resilience and coping strategies and supportive environments, highlighting how positive relationships and support, as well as everyday adaptive practices, can transform trajectories for young people with 22q.

#### 3.5.1. Supportive Professionals: Listening, Acting and Adapting

Supportive professionals, those who listened, acted decisively, and respected independence, enabled young people with 22q to flourish. Families consistently emphasised the difference made by professionals who went “*above and beyond*”, offering not only practical assistance but emotional reassurance. Proactive professionals helped dismantle barriers. For example, Maggie’s parent reflected on the advocacy provided during a funding dispute with the local authority: “*It was good to have somebody who was familiar with the SEN tribunal courts… She wrote a stinker letter to Surrey [council] saying, actually, you know, this is basically what you’re doing is illegal… And they wrote back and apologised.”* Similarly, social workers were praised for decisive action that prioritised wellbeing over bureaucracy. When Sarah was in a placement that was not suitable for her, *“The social worker pulled the plug on it and said, that’s it, she’s not getting any more. So, she got me another placement called Youth Inspired”* (Sarah’s parent).

Continuity of care was a rare but invaluable source of stability. Anna’s parent explained, *“She had the same GP like for a long, long time and he really knew her well… It was nice going in to talk to somebody and not having to tell her story over and over again.”* Thomas’s family echoed this sentiment, describing a GP who consistently prioritised their needs: *“He was always the one doctor that I knew I could go to… He understood [Thomas’] syndrome.”* Such relationships reduced emotional burden and fostered trust, enabling families to feel supported rather than isolated.

Professionals who adapted their approach to individual needs were seen as instrumental in fostering confidence and independence. Thomas’s experience in employment illustrates this: *“The manager in the kitchen was brilliant… He said if you just want to take time out, you want to go and step aside for 10–15 min, you do that and you just take as much time as you need.”* Similarly, Anna’s work coach was praised for her personalised support: *“She praises the small things… She’s personally taking her over to the volunteer center… She’s putting into place all the support that A will need to be successful.”*

Families also expressed relief when professionals demonstrated genuine expertise and collaborative thinking. Cara’s parent reflected on the impact of a new psychiatrist: *“The difference was she was actually prepared to listen to us… There was like this joined-up thinking rather than just ignore the parents.”* Maggie’s parent described the proactive stance of the paediatric 22q team: *“They’re probably going over and above… acting as a conduit between us and gastrology… I’m really grateful for that.”*

Supportive professionals were also key in fostering confidence and supporting milestones. Thomas’s parent reflected, *“For college, the tutors were lovely… They talked him through it and said it’s OK to struggle… And he finished the course and passed.”* Similarly, Maggie’s parent noted, *“Having this work coach is really helpful… I’ve got every confidence that this is going to work. I’m just going to end up in employment.”* After positive experiences with a new psychiatrist who took the time to understand his condition and treated his mental health appropriately, Peter reflected, *“It feels a lot better being happy and excited and looking forward to things.”*

#### 3.5.2. Supportive Places, Networks and Activities

Beyond navigating systemic gaps, supportive environments and networks enabled young people with 22q to flourish. Here, inclusive spaces, structured activities, and meaningful relationships fostered confidence, independence, and wellbeing.

Specialist and inclusive settings were repeatedly described as transformative. Maggie’s parent reflected on the impact of a residential college: *“It felt like a family… She was comfortable and relaxed.”* The college provided not only vocational training but also emotional safety: *“They worked on self-esteem… knowledge around consent… social communication classes… They were very good at keeping you up to date and supporting where they could see she might be struggling.”* Similarly, Sarah’s experience at ‘Youth Inspired’ [community organisation] highlighted the importance of respect and responsiveness: *“They listened to the young adult rather than listening to me* … *They didn’t wait for the anxiety to kick in… They just dealt with it there.”* Supportive places also enabled practical skill-building. Maggie’s parent highlighted the college’s focus on independence: *“They learn living independent skills… They worked in the hotel… all about improving their self-esteem and giving them strategies to cope once they left.”* Anna’s experience at ‘Horizons’ [youth centre] highlights her progress. While she was too unwell to fully engage initially, they agreed for her to come back and said, *“she did engage with the programme… She started to be able to go on the bus on her own… There was a lot she’d achieved.”* Anna herself reflected on her determination: *“I’ve been trying to push myself with my confidence”,* as well as wanting to *progress, “I want to do some volunteering before I have an apprenticeship or a job.”*

Families emphasised the role of networks in reducing isolation and promoting belonging. Thomas’s parent belonged to a workplace disability network, and they emphasised how helpful that support had been. Family support was also frequently described as invaluable. Peer relationships and friendships for young people were vital for them to thrive. Maggie’s parent noted the comfort of shared experiences: *“There was lots of young people like [her]… They all had commonality in that they all had learning disabilities… It felt like a family.”* Such friendships offered validation and companionship. Engagement in meaningful activities was linked to confidence and independence. Maggie’s parent described her much she enjoyed her voluntary work, and Maggie herself expressed excitement about new opportunities: *“I’m just excited about it… I’ve got friends up there as well that I know.”* Friendships were repeatedly described as lifelines. For Sarah, having a friend who truly understood her and who she felt she could talk to was important: *“Nathan helped me… He understood me.”* Similarly, Maggie spoke about the comfort of long-standing relationships, and Peter reflected on his best friend from his school days, stating that *“we can be away for months and we’re still exactly the same”.* These friendships also offered support towards independence.

Engagement in hobbies was often portrayed in the context of a coping strategy and a source of identity. Sarah explained how creative activities helped her regulate emotions: *“Painting… relaxed”* and *“Gaming… keeps me calm.”* Peter found photography to be a way of connecting with the world: *“I like doing photography… it’s nice to get out and go to events.”* Laura highlighted music and preparation strategies as tools for managing anxiety. Parents also emphasised the value of structured creative activities, such as drama and art classes, which provided routine and opportunities for self-expression: *“She’s [M] involved in a drama group… they’re doing a theatre presentation at Waterloo… She’s got involved in an art class and a trampolining group… quite a full timetable” (Maggie’s parent).* Maggie added she and her group “*would go out bowling and cinema… have discos for the birthdays and [she’s] going to Centre Parks.”*

This final superordinate theme illustrates how supportive relationships and inclusive environments enable resilience and wellbeing, pointing to relational and contextual factors that mitigate systemic shortcomings.

## 4. Discussion

This study explored the lived experiences of young people with 22q and their families as they navigated health and mental health support within the UK, including a focus on the transition to adulthood. Using a PAR framework, this study prioritised the voices of young people and their parents. Overall, the findings revealed experiences marked by limited awareness of 22q amongst professionals in the UK, fragmented systems, rigid mental health provision, and abrupt discontinuity during transition. However, enabling factors such as supportive professionals and relationships, as well as inclusive environments, highlighted key contributors to building confidence and wellbeing. While some over-arching themes identified in this study (e.g., fragmented care and parental advocacy) mirror patterns observed in other rare diseases (McMullan et al., 2020; Walton et al., 2023), the findings extend these in several important ways. They provide UK-specific evidence of systemic gaps for 22q noted internationally, including the absence of adult-focused 22q services, abrupt discharge from paediatric care, and reliance on generic mental health provision within NHS structures (Goodwin et al., 2020; O’Donoghue et al., 2022).

### 4.1. Summary of Key Findings

The themes revealed that one of the biggest barriers families encountered was a lack of recognition and appropriate support of 22q and its associated health and mental health complications, aligning with the findings found internationally (Ayoub et al., 2024; Carrion et al., 2022). The findings highlighted that this was often worsened due to lack of visible symptoms, and UK families resorted to using more familiar diagnoses (e.g., autism) to access support and services. When families did manage to access services, these were often generic and poorly adapted to the needs of 22q individuals, lacking joined-up support, and interactions with professionals were often experienced as dismissive. Mental health services were rigid, generic, and poorly adapted to 22q needs, echoing and extending findings by Campbell et al. (2024) and Goodwin et al. (2017). At times, these experiences were detrimental to the young people, and several families resorted to seeking private care to secure appropriate support.

Consistent with previous research on fragmented care pathways (Goodwin et al., 2020; Vo et al., 2018), families described acting as their child’s care coordinators across multiple hospital appointments and services. Due to barriers faced by families, parents often had to take on the role of advocate for their child to ensure appropriate support and access to services. Despite their expertise in own child’s needs, parents were often dismissed, and many had to navigate bureaucratic barriers, seek private care, or even engage in legal action to ensure support. The emotional toll of these experiences included stress, guilt, and strained family relationships. This role imposed significant emotional and practical burdens, often compounded by poor communication and siloed practices.

Often, the move from paediatric to adult services was abrupt or poorly managed, despite this period often coinciding with increased mental health vulnerability. These findings aligned with the literature by identifying transition as a high-risk period for individuals with 22q (Gadancheva et al., 2023), as well as highlighting a lack of adult-focused services for 22q in the UK, leaving families feeling abandoned and unsupported. The abrupt discharge from services, absence of adult-specific 22q provision, and pressure to conform to normative expectations of independence reflect broader structural failures in transitional planning as seen internationally (Ayoub et al., 2024; Gadancheva et al., 2023; Kerin et al., 2020). The findings highlighted parental concerns surrounding future uncertainty around independence, finances, and living arrangements of their children. These gaps reflect wider challenges in coordinating complex care. Evidence from acute settings shows that fragmented pathways arise from the poor integration of medical and broader needs, with nursing data revealing that care demands often exceed what diagnoses predict (Cesare et al., 2025). This mismatch heightens coordination burden and disrupts continuity, echoing our finding that families act as default care coordinators when systems fail to capture multidimensional needs.

Despite several obvious systemic challenges, several supportive factors of wellbeing were identified. Families described resilience and growth enabled by supportive professionals, inclusive environments, and strong social networks. Structured activities, hobbies, and friendships were also fundamental, helping young people with 22q build confidence, independence, and emotional stability. This aligns with research emphasising the protective role of relational and environmental factors in promoting wellbeing (Van de Woestyne et al., 2022), highlighting a need to consider resilience and enabling environments (Campbell et al., 2024).

### 4.2. Implications for Practice: Collaborative and Holistic Models

Combined, these findings support a need for participatory approaches that centre the voices of young people and their caregivers in shaping policy and practice (Goodwin et al., 2020; Jayman et al., 2025). In line with the PAR framework, narratives illustrate how families are not only recipients of care but also active agents navigating and resisting systemic gaps, offering critical insight into what meaningful support should look like for 22q. This reinforces this study’s central aim of exploring the lived experiences of UK families navigating mental health and transitional care.

The findings from this study are grounded in participant priorities and can inform systemic change at both service and policy levels. Key recommendations include specialist training for health and mental health professionals to improve early recognition of 22q and its psychiatric risks, supported by tailored modules for NHS clinicians, mental health practitioners, and educational staff. Dedicated care coordination roles within NHS trusts are essential to provide a single point of contact for families, reducing the current reliance on parents as informal coordinators and mitigating emotional burden. Nationally implemented transition protocols should ensure continuity between paediatric and adult services through joint appointments, sustained mental healthcare, and family involvement. Updated international guidelines reinforce the need for adult-focused, multidisciplinary care and specialist clinics to address gaps post-transition, aligning UK practice with global standards and reducing fragmentation (Boot et al., 2023). Finally, inclusive community-based programmes should promote peer networks, resilience building, and supported employment tailored to neurodevelopmental and genetic conditions.

These recommendations move towards providing concrete steps for improving care pathways, reducing advocacy burden, and supporting wellbeing across the lifespan. By embedding co-produced insights into policy and practice, these actions aim to create a more responsive, equitable system for individuals with 22q and their families.

### 4.3. Strengths, Limitations, and Future Directions

The PAR framework used strengthened this study by ensuring relevance and accessibility through co-developed interview guides and iterative interpretation, revealing nuanced experiences, specific to this study, such as the strategic use of alternative diagnoses to access support and the emotional deterioration linked to normative expectations of independence during transition. The experiences described by families emphasise the importance of co-produced resources that amplify family expertise and challenge assumptions embedded in current UK practice, ensuring parental expertise is validated rather than dismissed. By incorporating young people’s perspectives through PAR, this study responds to calls for participatory frameworks in rare disease research (Ayoub et al., 2024), addressing a critical gap in representation and ensuring findings reflect lived priorities rather than solely parental or clinical views.

Some limitations are to be noted. This study was UK-specific, which may affect overall generalizability. It lacked ethnic diversity, further limiting the transferability of findings to families from minority backgrounds who may experience additional cultural or systemic barriers. This study included a relatively small sample size, which is, however, appropriate for a PAR study and in line with similar studies, prioritising depth and co-production over breadth, allowing for rich, detailed narratives (Braun & Clarke, 2006; Swain, 2018). While there was potential for self-selection bias in this study, as families with a particular interest in or experience with 22q might have been more likely to participate, informal feedback suggested non-participation was mainly due to practical constraints. In addition, although interviews provided rich narratives, they relied on retrospective accounts, introducing potential recall bias. This may have influenced how participants remembered and framed their experiences. Future research should prioritise inclusive recruitment strategies and longitudinal or real-time data collection to help mitigate this limitation in future studies. Finally, while excluding professionals’ perspectives was a deliberate choice to centre family voices, it represents an important avenue for future research.

These findings advance the understanding of service navigation for 22q in the UK, moving beyond descriptive accounts toward actionable insights for policy and practice. Further research should explore adult services and long-term outcomes, incorporate professionals’ perspectives to inform systemic change, and evaluate co-designed, personalised mental health interventions for mental health and transition support informed by lived experience.

## 5. Conclusions

This study, to the best of our knowledge, was the first to explore how families affected by 22q navigate health and mental health systems in the UK and experience transition to adulthood, through a participatory action research (PAR) framework. The findings identified systemic barriers, such as lack of professional awareness, fragmented care pathways, and abrupt discontinuity during transition, and highlighted enabling factors, including supportive professionals, inclusive environments, and peer networks. By centring family and young adult voices in both question development and interpretation, this study demonstrates the value of co-production in generating actionable recommendations for practice, such as specialist training, structured transition planning, and integrated care coordination—many of which are also highly relevant to other complex genetic conditions. These insights advance the understanding of service navigation for 22q in the UK and illustrate how participatory approaches can inform responsive, holistic models of care.

## Figures and Tables

**Table 1:** Participant characteristics.

Pseudonym	Age	Gender
Cara	31	Female ^1^
Peter	30	Male
Anna	24	Female
Sarah	23	Female
Maggie	23	Female
Thomas	22	Male

^1^ Only the parent took part.

**Table 2:** Example questions from semi-structured interviews as informed by the steering group.

Topic Area	Example Question
Awareness and Context: Explored perceptions of professional understanding and priorities.	Do you believe that emotional wellbeing is a priority for professionals working with your child? Why or why not?
Support and Barriers: Focused on practical experiences of accessing care.	Has your child accessed any mental health services or treatments recently? If so, were there any barriers (e.g., waiting times, online vs. in-person, lack of specialist provision)?
Transition Experiences and Communication—including suggestions for systemic improvement.	Do you have any suggestions for improving the systems of support in place for your child’s mental wellbeing during transitions?

**Table 3:** Example citations from the literature.

Author	Example Citation
Vo et al. (2018)	*“Families report significant psychosocial burdens and systemic challenges in accessing appropriate care” p. 2216*
Carrion et al. (2022)	*“Parents report limited professional understanding of 22q11.2DS, contributing to diagnostic delays” p. 140*
Gadancheva et al. (2023)	*“Time of transition is particularly vulnerable; additional focus on planning will improve outcomes for individuals with 22q11.2DS and their families” p. 716*
Campbell et al. (2024)	*“Existing literature has a heavy focus on delineating psychiatric comorbidities but very few studies explore how to adapt and implement effective interventions to support mental health and well-being in 22q11.2DS” p. 2*

**Table 4:** Braun and Clarke’s six-phrase framework for thematic analysis.

Author	Example Citation
Familiarisation with the data	All interviews were transcribed verbatim and reviewed by the research team. Each transcript was read and re-read to ensure deep immersion, and audio recordings were revisited where necessary to clarify tone, context, or meaning. Initial observations and potential patterns were noted independently by the researchers.
Generating initial codes	Each researcher independently coded the transcripts using both descriptive and ‘in vivo’ code, terms and phrases used directly by participants. Coding was conducted manually to maintain close engagement with the data and subsequently collated into a shared document with illustrative excerpts attached to each. Both deductive and inductive codes were applied.
Searching for themes	Authors compared and discussed initial codes, identifying areas of overlap and divergence, codes were grouped into broader categories, and meaningful themes and subthemes were developed collaboratively, reflecting both conceptual categories and emergent patterns.
Reviewing themes	Themes were reviewed against the full dataset to ensure they accurately reflected participant narratives and were refined, merged, or discarded to ensure meaningful and comprehensive interpretation of the dataset.
Defining and naming themes	Themes were clearly defined and labelled to capture their essence in a concise and meaningful way. Subthemes were identified to reflect nuanced aspects of participants’ experiences while remaining grounded in guiding concepts.
Producing the report	The final stage involved writing up the analysis, integrating thematic narratives with illustrative quotes. The write-up aimed to present a coherent and compelling account of participants’ experiences, as a product of both the research question and the authors’ prolonged immersion of the data and continuous reflection. Reflexivity was maintained throughout to ensure the research team critically reflected on positionality and influence on interpretation.

## Data Availability

The original contributions presented in this study are included in the article. Further inquiries can be directed to the corresponding author.

## Abbreviations

The following abbreviations are used in this manuscript:

22q	22q11.2 Deletion Syndrome
CAMHS	Children and Adolescent Mental Health Services
PAR	Participatory Action Research
GP	General Practitioner
SENCO	Special Educational Needs Coordinator
LD	Learning Disability
A&E	Accident and Emergency
NHS	National Health Service
